# Foundation Model Based on Routine Magnetic Resonance Imaging for Brain Tumor Molecular Profiling and Progression Prediction

**DOI:** 10.1200/PO-25-00930

**Published:** 2026-02-20

**Authors:** Junxian Li, Renhe Liu, Yuchen Xing, Ximin Gao, Qiang Yin, Qian Su

**Affiliations:** ^1^ Department of Blood Transfusion, Key Laboratory of Cancer Prevention and Therapy, Tianjin, National Clinical Research Center for Cancer, Tianjin's Clinical Research Center for Cancer, Tianjin Medical University Cancer Institute & Hospital, Tianjin Medical University, Tianjin, China; ^2^ Department of Diagnostic and Therapeutic Ultrasonography, Key Laboratory of Cancer Prevention and Therapy, Tianjin, National Clinical Research Center for Cancer, Tianjin's Clinical Research Center for Cancer, Tianjin Medical University Cancer Institute & Hospital, Tianjin Medical University, Tianjin, China; ^3^ Department of Cancer Epidemiology and Biostatistics, Key Laboratory of Molecular Cancer Epidemiology, Tianjin, National Clinical Research Center for Cancer, Tianjin's Clinical Research Center for Cancer, Tianjin Medical University Cancer Institute & Hospital, Tianjin Medical University, Tianjin, China; ^4^ Department of Neurosurgery and Neuro-oncology, Key Laboratory of Cancer Prevention and Therapy, Tianjin, National Clinical Research Center for Cancer, Tianjin's Clinical Research Center for Cancer, Tianjin Medical University Cancer Institute & Hospital, Tianjin Medical University, Tianjin, China; ^5^ Department of Molecular Imaging and Nuclear Medicine, Key Laboratory of Cancer Prevention and Therapy, Tianjin, National Clinical Research Center for Cancer, Tianjin's Clinical Research Center for Cancer, Tianjin Medical University Cancer Institute & Hospital, Tianjin Medical University, Tianjin, China

## Abstract

**PURPOSE:**

To build a self-supervised magnetic resonance imaging (MRI) foundation model from routine clinical scans and to test whether it can support key glioma-related applications, including post-therapy imaging outcome characterization and molecular marker inference.

**MATERIALS AND METHODS:**

We created the Unified Multimodal Brain Imaging Foundation (UMBIF) model and pretrained it in a self-supervised manner using 51,029 routine brain MRI examinations collected across multiple institutions. Pretraining used a hybrid objective that couples masked-image reconstruction with contrastive representation learning to encourage anatomically and clinically informative embeddings. The pretrained UMBIF encoder was then adapted to downstream multicenter data sets to predict (1) post-treatment radiographic outcomes and (2) molecular biomarkers, including *IDH *mutation, *MGMT* promoter methylation, and 1p/19q codeletion. Performance was benchmarked against commonly used convolutional networks and traditional machine learning classifiers, using accuracy, sensitivity, specificity, and receiver operating characteristic-AUC as primary metrics.

**RESULTS:**

Relative to self-supervised initialization derived from natural-image corpora or from approaches emphasizing only large tumor-area crops (self-supervised learning [SSL]-ImageNet and SSL-Cerebral), the UMBIF encoder-decoder design captured richer, more task-relevant features and consistently improved downstream discrimination. The best pretrained model achieved an accuracy of 0.899 (AUC, 0.815) for post-treatment radiographic outcome characterization. For molecular profiling, it reached accuracies/AUCs of 0.898/0.916 for 1p/19q codeletion, 0.829/0.896 for *IDH* mutation status, and 0.905/0.859 for* MGMT* promoter methylation, indicating strong potential utility in clinical decision support.

**CONCLUSION:**

UMBIF showed robust transferability to both post-therapy imaging assessment and molecular status prediction in glioma. By leveraging large-scale self-supervised pretraining to boost performance while reducing dependence on manual annotations, the framework may facilitate more efficient and reliable diagnostic workflows.

## INTRODUCTION

Gliomas are the most frequent malignant primary brain tumors, with substantial molecular diversity and heterogeneous prognostic outcomes. Molecular biomarkers, including *IDH* mutation, 1p/19q codeletion, and *MGMT *promoter methylation, inform diagnosis, prognosis, and post-treatment radiographic assessment.^1^ These markers are determined from tumor tissue, and even a diagnostic biopsy carries risk when resection is not feasible, motivating noninvasive methods that approximate tissue-based diagnosis.

**CONTEXT**

**Key Objective**
Can a self-supervised foundation model trained on routine T1-weighted contrast-enhanced magnetic resonance imaging (MRI) enable accurate multicenter glioma molecular profiling and post-treatment radiographic outcome characterization while reducing annotation dependence versus existing approaches?
**Knowledge Generated**
Unified Multimodal Brain Imaging Foundation model pretrained on 51,029 MRIs and fine-tuned on multicenter cohorts outperformed self-supervised learning baselines (ImageNet, single-region). Achieved accuracies/AUCs: post-treatment radiographic outcome characterization 0.899/0.815; 1p/19q codeletion 0.898/0.916; *IDH* mutation status 0.829/0.896; *MGMT* promoter methylation 0.905/0.859; and DeLong *P* < .05 where tested.
**Relevance**
By providing a scalable MRI-based self-supervised foundation model that accurately predicts post-treatment radiographic outcome and key molecular biomarkers in glioma, this study offers a practical decision-support tool to personalize therapy, guide follow-up, and potentially reduce the need for invasive testing and extensive manual annotation in routine clinical practice.

Magnetic resonance imaging (MRI) is the mainstay for glioma diagnosis and for tracking treatment response, owing to its noninvasive nature and excellent soft tissue contrast.^2^ Routine sequences (T1-weighted imaging, T2-weighted imaging, fluid-attenuated inversion recovery, and T1-weighted contrast-enhanced imaging [T1CE]) capture anatomic alterations, whereas diffusion-weighted imaging and magnetic resonance spectroscopy offer insight into tumor microstructure and metabolism.^3^ However, interpretation still depends largely on radiologists' subjective evaluation, which constrains accurate prediction of glioma molecular characteristics.

To overcome limitations of conventional MRI interpretation, artificial intelligence (AI), particularly deep learning (DL), is used to extract clinically relevant information. DL models can noninvasively predict key biomarkers, including *IDH* mutation, 1p/19q codeletion, and *MGMT* promoter methylation,^4,5^ and, with radiogenomics, model post-treatment radiographic outcomes.^6,7^ Current approaches mainly combine radiomics-based machine learning (ML) with DL-based feature extraction: radiomics relies on handcrafted features that are sensitive to scanner and protocol variation, whereas DL learns device-independent representations from images. Yet DL still demands large labeled data sets, and models often generalize poorly across institutions and acquisition protocols, underscoring the need for more robust, scalable AI in glioma care.

The large volume of unlabeled data available in clinical settings creates a pathway to alleviate the scarcity of annotated samples required by supervised learning. Self-supervised learning (SSL) has become a central strategy, allowing models to acquire transferable representations through pretext tasks such as contrastive learning or masked image modeling. These representations can then be fine-tuned for targeted medical applications, including tumor segmentation, radiology anomaly detection, and histopathology analysis, thereby decreasing dependence on large labeled data sets. Vision foundation models pretrained with SSL have improved cancer imaging biomarker discovery,^8^ retinal and systemic disease prediction,^9^ and gastrointestinal endoscopy analysis,^10^ enhancing robustness when labels are limited. Because medical and natural images differ, such models require domain-specific adaptation through fine-tuning on medical data, lightweight adapters, prompting, or specialized architectures to become clinically useful.

Building on our previously published Unified Multimodal Brain Imaging Foundation (UMBIF) model,^11^ this study advances the framework from an initial foundation-model demonstration to a more comprehensive and clinically oriented evaluation setting. UMBIF adopts the Masked Autoencoder scheme and is pretrained on an unlabeled multimodal brain MRI cohort of 2,312 patients, learning multisequence whole-brain volumetric representations rather than tumor-only patches. On this basis, we implement task-specific fine-tuning for classification of *IDH* mutation, 1p/19q codeletion, *MGMT* promoter methylation, and post-treatment radiographic outcomes. Compared with our previous report, we further emphasize cross-cohort validation and robustness/generalizability assessment, and benchmark against strong DL and ML baselines using multiple performance metrics.

## MATERIALS AND METHODS

### Data Set Sources and Patients

#### Data Sets for Constructing the UMBIF Encoder-Decoder Model

We curated 13 glioma-associated MRI data sets from The Cancer Imaging Archive (TCIA; N = 1,321 patients) and 119 Kaggle data sets spanning glioma, Alzheimer's disease, and dementia. To avoid data leakage, MRI scans later used for downstream evaluations were removed. The remaining data sets were aggregated to form the pretraining cohort. Full details of the pretraining data sets are reported in the Data Supplement (Tables S1 and S2), and all data are publicly accessible.

#### Downstream Clinical Task Data Sets

To assess performance in post-treatment radiographic outcome characterization, specifically differentiation of pseudoprogression (PsP) from tumor progression (TuP), we used the Burdenko-GBM-Progression data set and The University of Pennsylvania Health System's multiparametric MRI data set for patients with glioblastoma (UPENN-GBM), yielding 161 MRI scans.

To assess prediction of genetic and molecular characteristics, we used Burdenko-GBM-Progression, The Brain Resection Multimodal Imaging Database (ReMIND), and The Cancer Genome Atlas Low-Grade Glioma Collection (TCGA-LGG). Based on molecular data, patients were grouped by* MGMT* promoter methylation (methylated *v* unmethylated) and* IDH* mutation status (*IDH*-mutant *v* *IDH*–wild-type). This classification yielded 1,100 MRI scans for *IDH* status and 485 scans for *MGMT* promoter methylation.

For 1p/19q codeletions analysis, we used the ReMIND and TCGA-LGG data sets. Patients were classified into two groups—those with and those without the codeletion—based on clinical and molecular information. A total of 485 MRI scans were extracted for this analysis. By restricting to patients with complete imaging and molecular data, we ensured a robust basis for evaluating molecular prediction across different glioma subtypes. The Data Supplement (Table S3 and I) summarizes the downstream-task data sets and indicate their public accessibility in detail.

### Data Preprocessing

For pretraining, we prepared two input types in parallel: whole-brain 3D volumes and, for each patient, a single 2D slice capturing the maximal region of interest. This design allowed a head-to-head comparison of these representations during the first-stage training. The details of the data preprocessing are shown in the Data Supplement (II).

### UMBIF Encoder-Decoder Architecture and Training

To facilitate self-supervised visual representation learning, we developed a 3D medical-imaging autoencoder based on a Vision Transformer (ViT; UMBIF encoder-decoder). The framework includes two modules: a ViT encoder and a transformer decoder. In pretraining, the encoder ingested masked patch tokens and produced latent feature embeddings for the underlying MRI volumes. The decoder then combined the encoder outputs with mask tokens to recover the omitted patches under a masked autoencoding objective. This design enables learning informative visual representations without requiring manual labels. Additional architectural details and evaluation metrics are provided in the Data Supplement (III).

We evaluated the UMBIF encoder against two pretrained reference models: SSL-ImageNet and SSL-Cerebral. Although their pretraining paradigms differed, they used the same backbone architecture and an identical fine-tuning scheme. As illustrated in the Data Supplement (Fig S1), SSL-Cerebral retains the same network design for both pretraining and downstream fine-tuning. By contrast, the UMBIF encoder-decoder follows a hybrid pipeline: it is initialized with SSL-ImageNet weights and then further pretrained on brain MRI, corresponding to sequential SSL pretraining on natural images followed by brain images. A schematic summary of these pretraining routes is provided in the Data Supplement (Fig S2).

**FIG 1.**
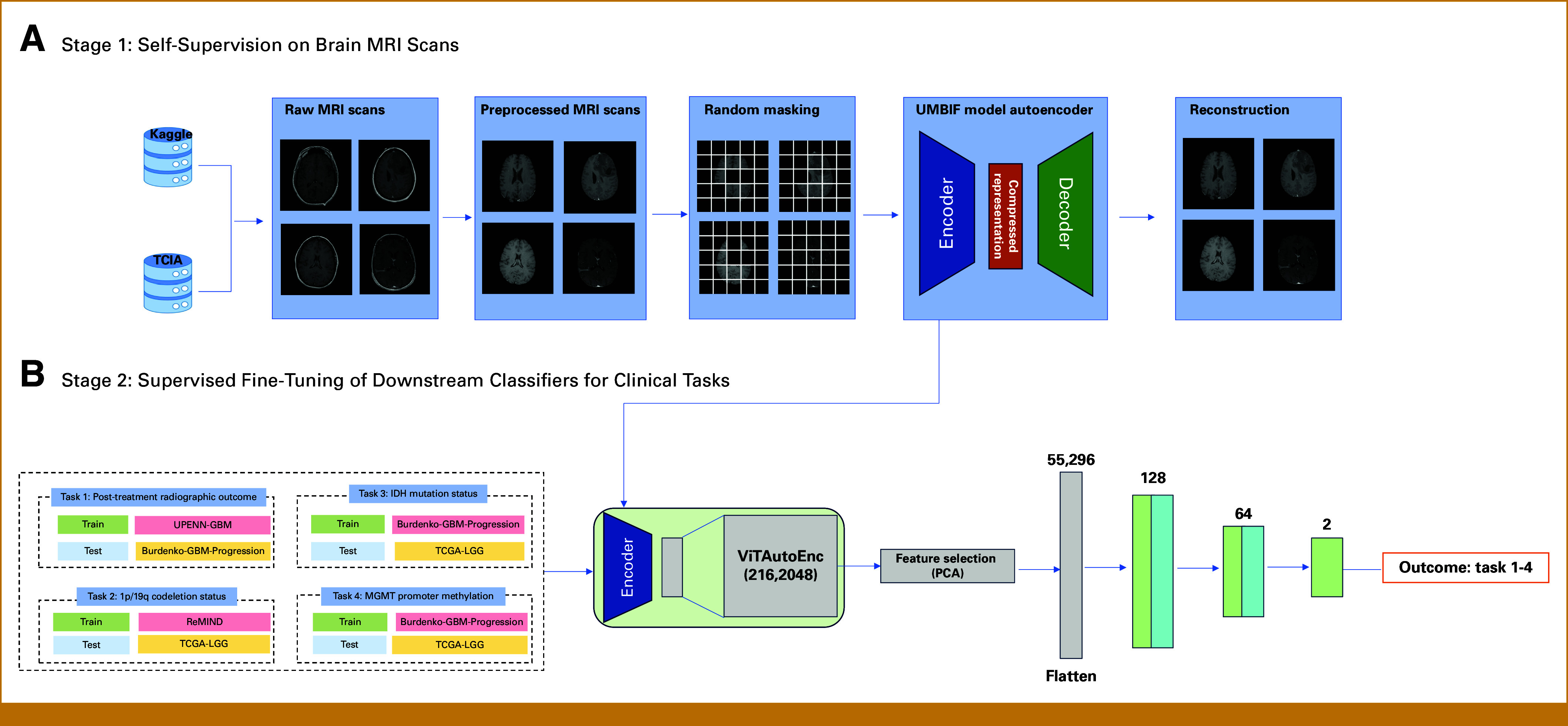
The development and assessment process of the foundation models (UMBIF) follows a structured approach. (A) In the initial phase, UMBIF encoder-decoder is built through SSL, incorporating T1WI, T2WI, T1CE, and FLAIR data sourced from TCIA and Kaggle repositories. (B) The subsequent phase involves refining UMBIF encoder for specific downstream applications, utilizing supervised learning to facilitate both internal validation and external performance evaluation. FLAIR, fluid-attenuated inversion recovery; MRI, magnetic resonance imaging; PCA, principal component analysis; ReMIND, The Brain Resection Multimodal Imaging Database; T1CE, T1-weighted contrast-enhanced imaging; T1WI, T1-weighted imaging; T2WI, T2-weighted imaging; TCIA, The Cancer Imaging Archive; TCGA-LGG, Cancer Genome Atlas Low-Grade Glioma Collection; UMBIF, Unified Multimodal Brain Imaging Foundation; UPENN-GBM, The University of Pennsylvania Health System's multiparametric MRI data set for patients with glioblastoma.

Throughout training, we tracked validation-set reconstruction loss to identify and save the optimal model weights. The complete UMBIF development pipeline is shown in Figure 1A.

### Classifier Development and Comparison Algorithms

For downstream classification, we appended a classification head to the pretrained ViTAutoEnc. This head includes a feature-selection layer and a stack of fully connected layers to highlight the most informative representations (Fig 1B, Data Supplementary, Ⅳ).

Three convolutional neural networks (CNN)—ResNet-34,^12^ DenseNet-121,^13^ and ShuffleNet^14^—were pretrained on ImageNet1k and implemented via the timm (PyTorch Image Models) library. Second, we used features extracted from these CNNs to construct and evaluate a variety of traditional ML classifiers. Detailed information about the CNNs and ML algorithms used in this study can be found in the Data Supplement (V and VI).

To evaluate scalability of the pretrained UMBIF encoder, we designed four binary classification tasks: task 1, distinguishing PsP from TuP after chemoradiotherapy; task 2, assessing 1p/19q codeletion status in gliomas; task 3, predicting *IDH* mutation status in gliomas; and task 4, determining *MGMT* promoter methylation status in gliomas.

### Statistical Analysis

We used the DeLong test to assess differences in AUC between classification models, implemented in the pROC package (an R package for receiver operating characteristic curve analysis) in R (version 4.3.2). *P* value <.05 was considered statistically significant.

### Ethical Approval

This study was conducted in accordance with the Declaration of Helsinki and was approved by the Ethics Committee of Tianjin Medical University Cancer Hospital (approval number: EK20240287).

### Informed Consent

Written informed consent was waived by the institutional review board.

## RESULTS

### Characteristics of the Data

We adopted a two-phase training strategy to construct a conventional MRI–based brain analysis model for multiple clinical end points. In stage one, the UMBIF encoder-decoder was pretrained on N = 51,029 images (n = 40,823 for training; n = 10,206 for testing) to learn robust feature representations. In stage two, we performed supervised fine-tuning on multi-institutional T1CE data to evaluate generalizability across cohorts. Table 1 summarizes the data sets used for training and testing. For post-treatment radiographic outcome characterization, UPENN-GBM was used for training and Burdenko-GBM-Progression for testing. For 1p/19q codeletion prediction, ReMIND and TCGA-LGG served as the training and testing data sets. Burdenko-GBM-Progression and TCGA-LGG were used for *IDH* mutation and *MGMT* promoter methylation prediction. Detailed image counts for all cohorts are provided in Table 1.

**TABLE 1.** Comprehensive Count of Images Used in Each Task

Task Descriptions	Training Data Set	Testing Data Set	Aggregate Total
Stage 1: SSL on brain magnetic resonance images	40,823	10,206	51,029
Stage 2: Supervised fine-tuning for clinical tasks			
Task 1: Post-treatment radiographic outcome characterization			
PsP	8	33	41
TuP	34	86	120
Task 2: 1p/19q codeletion status prediction			
Intact	262	71	333
Codeleted	121	31	152
Task 3: *IDH* mutation status prediction			
Wild-type	635	78	713
Mutant	79	308	387
Task 4: *MGMT* promoter methylation			
Unmethylated	57	76	133
Methylated	37	315	352

Abbreviations: MRI, magnetic resonance imaging; PsP, pseudoprogression; SSl, self-supervised learning; TuP, tumor progression.

### Performance Assessment of UMBIF Encoder Model for 1p/19q Codeletion Status and Post-Treatment Radiographic Outcome Characterization

**FIG 2.**
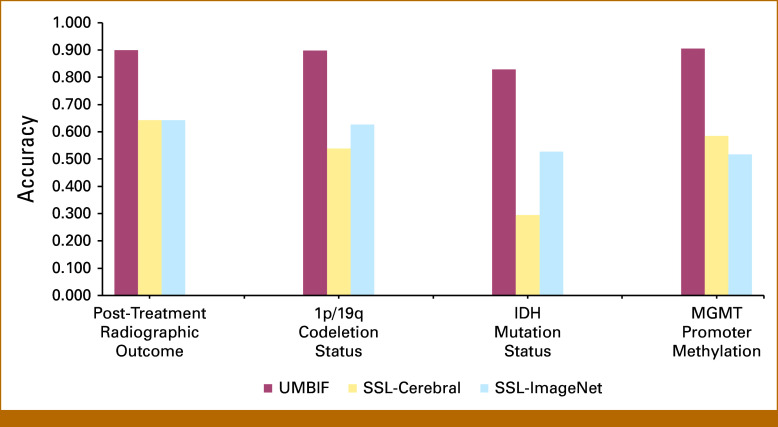
Performance comparison of UMBIF encoder and SSL-based methods across classification tasks. Across all tasks, UMBIF encoder consistently outperformed the SSL-based methods, achieving higher accuracy in all categories. SSL, self-supervised learning; UMBIF, Unified Multimodal Brain Imaging Foundation.

**FIG 3.**
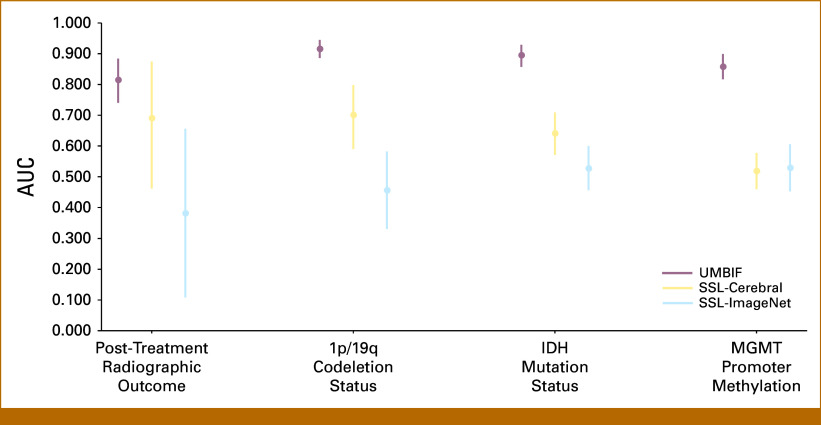
Comparison of AUC performance between UMBIF encoder and SSL-based methods. The figure presents the AUC with 95% CI for UMBIF, SSL-Cerebral, and SSL-ImageNet across multiple classification tasks. UMBIF encoder consistently achieved higher AUC values compared with SSL-based approaches, indicating superior predictive performance. The CIs for UMBIF encoder are also narrower in several tasks, suggesting greater model stability. SSL, self-supervised learning; UMBIF, Unified Multimodal Brain Imaging Foundation.

The UMBIF encoder was evaluated for post-treatment outcome characterization and 1p/19q codeletion status and showed superior performance across data sets. For 1p/19q codeletion, it achieved an accuracy of 0.898 (Fig 2) and an AUC of 0.916 (95% CI, 0.886 to 0.945; Fig 3, *P* = .001), outperforming SSL-Cerebral and SSL-ImageNet. For post-treatment radiographic outcome characterization, the encoder reached an accuracy of 0.899 and an AUC of 0.815 (95% CI, 0.740 to 0.885; Fig 3, *P* = .001), again exceeding SSL-based methods (Fig 2). On the Burdenko-GBM data set, sensitivity, positive predictive value (PPV), negative predictive value (NPV), and F1 score were 0.899, 0.990, 0.878, and 0.778, respectively, indicating balanced detection and predictive accuracy. By contrast, SSL-Cerebral achieved a sensitivity of 0.875 but lower specificity (0.588), PPV (0.333), and F1 score (0.483), whereas SSL-ImageNet showed sensitivity and F1 scores of only 0.250 and 0.211, respectively (Table 2).

**TABLE 2.** Performance Comparison of Different Algorithms on Clinical Tasks of Varying Difficulty in Testing Data

Outcome	UMBIF					SSL-Cerebral					SSL-ImageNet				
	Sensitivity	Specificity	PPV	NPV	F1	Sensitivity	Specificity	PPV	NPV	F1	Sensitivity	Specificity	PPV	NPV	F1
Post-treatment radiographic outcome	0.899	0.636	0.990	0.878	0.942	0.875	0.588	0.333	0.643	0.483	0.250	0.735	0.182	0.806	0.211
1p/19q codeletion status	0.711	0.985	0.956	0.881	0.815	0.936	0.366	0.392	0.660	0.552	0.226	0.803	0.333	0.704	0.269
*IDH* mutation status	0.847	0.756	0.932	0.557	0.888	0.127	0.962	0.929	0.573	0.223	0.506	0.615	0.839	0.240	0.632
*MGMT* promoter methylation	0.568	0.842	0.724	0.727	0.636	0.683	0.184	0.776	0.450	0.726	0.486	0.645	0.850	0.232	0.618

Abbreviations: NPV, negative predictive value; PPV, positive predictive value; SSL, self-supervised learning; UMBIF, Unified Multimodal Brain Imaging Foundation.

### Performance Assessment of UMBIF Encoder Model for Determining *IDH* Mutation Status and *MGMT* Promoter Methylation Status

UMBIF encoder achieved an *IDH* mutation classification accuracy of 0.829 on the TCGA-LGG data set (Fig 2), with an AUC of 0.896 (95% CI, 0.857 to 0.929). Accuracy of SSL-based methods was markedly lower (0.295 for SSL-Cerebral; Fig 2). Clinical evaluation in Table 2 showed specificity, PPV, and F1 scores of 0.756, 0.932, and 0.888, respectively, supporting* IDH* mutation classification. For *MGMT* promoter methylation classification, UMBIF encoder achieved an accuracy of 0.905 on TCGA-LGG, higher than SSL-based methods (best accuracy 0.586 for SSL-Cerebral; Fig 2). The AUC was 0.859 (95% CI, 0.817 to 0.900; Fig 3, *P* = .001). TCGA-LGG yielded a PPV of 0.724, an F1 score of 0.636, and an NPV of 0.727 (Table 2), confirming robust *MGMT* classification.

### Comparison of DL and ML Model Performance

The Data Supplement (Tables S4 and S5) report the comparative results of ML and DL approaches across the classification end points. Across these tasks, the UMBIF encoder achieved the top performance, with the best accuracy and AUC reported in the Data Supplement (Tables S4 and S5), underscoring its strong classification ability across diverse clinical settings.

## DISCUSSION

In this work, we built and validated UMBIF, an encoder-decoder SSL framework for brain MRI that limits dependence on manual annotations. Trained on a multi-institutional cohort of 51,029 magnetic resonance images, UMBIF acquired strong and transferable representations through cross-contrast context restoration and achieved state-of-the-art performance on neuro-oncologic classification tasks. Relative to self-supervised approaches pretrained on natural images or on single large tumor regions, UMBIF delivered improved classification accuracy. It also consistently exceeded widely used CNN baselines and traditional ML methods for post-treatment radiographic outcome characterization (AUC, 0.815), 1p/19q codeletion prediction (AUC, 0.916), *IDH* mutation status prediction (AUC, 0.896), and *MGMT* promoter methylation prediction (AUC, 0.859). Across all end points, UMBIF outperformed SSL-Cerebral and SSL-ImageNet, underscoring its robustness and translational potential.

Many groups have applied DL methods to build prediction models for a range of clinical end points, but overall performance is still suboptimal. Sun et al^15^ distinguished PsP from TuP in patients with GBM using T1CE, reporting an AUC of 0.730. By contrast, our approach yielded a higher AUC for post-treatment radiographic outcome characterization while covering a wider spectrum of downstream tasks. Choi et al^16^ used a ResNet-34 to infer *IDH* mutation status, achieving AUCs of 0.940 and 0.860 in two independent external validation cohorts. Hosseini et al^17^ used GAN-based data augmentation in patients with newly diagnosed grade 4 astrocytoma and GBM and achieved an AUC of 0.930, but the single-center, small-sample design constrained clinical applicability. Saxena's group^18^ built a ResNet-18 model to predict *MGMT *promoter methylation from multiparametric MRI, with an AUC of 0.753, where dependence on large data sets and the lack of high-quality MRI likely constrained further gains. Van der Voort's team^19^ proposed a multitask U-Net–based framework trained on nine sources to jointly perform molecular subtyping and grading, reaching AUCs of 0.850 (95% CI, 0.770 to 0.920) for 1p/19q codeletion and 0.900 (95% CI, 0.850 to 0.950) for *IDH* mutation status in TCIA cohorts. Direct cross-study comparisons remain challenging because imaging protocols, inclusion criteria, and evaluation schemes vary substantially. Relative to these efforts, our foundation model leverages extensive multimodal MRI and SSL pretraining to learn more generalizable representations, requiring only modest labeled data for adaptation across tasks ranging from GBM molecular profiling to PsP-TuP differentiation. Collectively, previous work underscores both the potential of DL-derived imaging biomarkers and ongoing barriers, including limited data diversity, constrained generalizability, heavy reliance on large labeled cohorts, and task-specific architectures that are difficult to reuse for new indications.

Insufficient access to high-quality annotated data continues to be a major constraint in medical image analysis. Traditional supervised learning depends on large quantities of manually labeled MRI scans, but the inherent complexity of medical imaging and substantial interinstitutional variation make annotation labor-intensive and costly. SSL provides an attractive solution by supporting large-scale pretraining on unlabeled data sets, enabling models to capture structured image features without direct task-specific supervision. This property is particularly useful when expert labels are limited or inconsistent across centers. In this work, we propose a cross-contrast context restoration objective that exploits SSL to learn generalizable brain imaging representations from large unlabeled cohorts, followed by fine-tuning to improve performance on downstream applications. Previous reports have also highlighted the utility of SSL in medical imaging. Tak's group^20^ demonstrated that SSL can markedly boost the classification performance of conventional supervised approaches for pediatric low-grade glioma. Cox et al^21^ further reported superior classification performance and robustness with SSL under heterogeneous and weakly labeled conditions. Extending these observations to neuro-oncology, our results support the effectiveness of SSL for post-treatment radiographic outcome characterization and molecular biomarker prediction.

A further important observation is the consistent performance of the UMBIF encoder-decoder across multicenter cohorts. Variations in MRI acquisition protocols, scanner hardware, and underlying patient characteristics commonly impede cross-site generalization. Conventional supervised models often achieve strong results on the development data set but suffer noticeable degradation when evaluated on external cohorts. By contrast, we assessed the robustness of the UMBIF encoder on several independent data sets, including ReMIND, TCGA, and Burdenko-GBM, and found that it consistently surpassed pretrained baselines such as SSL-Cerebral and SSL-ImageNet across all test sets—an important prerequisite for clinical deployment of AI systems. This pattern is in line with Wang et al,^22^ who showed that SSL pretraining can substantially improve cross-center generalization for pathology imaging models and reduce performance loss driven by distribution shifts. Taken together, our findings indicate that SSL pretraining not only alleviates the need for extensive annotations but also strengthens robustness under heterogeneous scanning environments.

In post-treatment radiographic outcome characterization, the UMBIF encoder achieved an accuracy of 0.899 on the Burdenko-GBM data set, outperforming baseline methods. This demonstrates that SSL can capture changes in the tumor microenvironment and automatically mine relevant imaging features related to post-treatment radiographic outcome characterization, offering a more efficient alternative to traditional radiomics methods. Similar findings were observed by Jin et al,^23^ who demonstrated that SSL could extract stable treatment response–related features from MRI scans, significantly improving the prediction of therapy response in patients with rectal cancer.

Regarding molecular biomarker prediction, the UMBIF encoder demonstrated excellent performance in predicting *IDH* mutation status, *MGMT* promoter methylation, and 1p/19q codeletion mutations, achieving accuracies of 0.829, 0.905, and 0.898, respectively. These results suggest that SSL can capture deeper radiogenomic associations, providing improved prediction performance over traditional radiomic methods. This aligns with Parmar et al,^24^ who demonstrated that SSL significantly improved the prediction accuracy of genetic biomarkers. Similarly, Shao et al^25^ found that contrastive SSL methods enhanced molecular biomarker prediction in colorectal cancer, outperforming traditional radiomics and ImageNet pretraining. Moreover, our study challenges the prevailing reliance on transfer learning from natural image data sets, showing that direct SSL pretraining on medical images leads to superior performance compared with models pretrained on ImageNet. These observations argue for SSL paradigms tailored to medical imaging.

The findings of this study have implications for both theory and clinical practice. From a theoretical perspective, this study provides a systematic evaluation of SSL's adaptability in multitask medical image analysis, showing that SSL can effectively capture imaging patterns from MRI and uncovering latent correlations between MRI data and tumor molecular states, enhancing the accuracy of neuro-oncology diagnosis. In molecular biomarker prediction tasks, UMBIF encoder achieved AUCs of 0.896 and 0.859, respectively, for *IDH* mutation status and *MGMT* promoter methylation prediction, outperforming SSL-Cerebral and SSL-ImageNet, extending SSL's applicability across multicenter data sets. From an applied perspective, UMBIF functions as a general-purpose medical imaging foundation model that can lessen radiologists' labeling workload and enable a more efficient, cost-conscious route toward precision medicine. It can be embedded within clinical decision-support pipelines to aid clinicians in preoperative assessment of post-treatment radiographic outcomes and in identifying major genetic alterations, thereby supporting more personalized treatment strategies for individual patients.

In clinical workflows, the UMBIF encoder is intended as an imaging-based decision-support adjunct rather than a replacement for histopathologic diagnosis. Preoperatively, it supports noninvasive stratification—including prediction of *IDH* mutation status, 1p/19q codeletion, and *MGMT* promoter methylation—to inform the choice of surgical/biopsy pathways and the planned extent and targets of sampling. During post-treatment surveillance (postoperative and during chemoradiation), it assists in distinguishing PsP from TuP and may reduce unnecessary invasive procedures. When tissue acquisition is contraindicated or poses substantial risk, the model can provide preliminary stratification and facilitate clinical-trial prescreening. Model outputs comprise patient-level predictions accompanied by calibrated uncertainty estimates and out-of-distribution alerts, and are reviewed in multidisciplinary team conferences alongside conventional imaging features and clinical data to support individualized treatment decisions. Implementation relies on standardized inputs and site-level calibration of decision thresholds and uncertainty reporting, and the system does not supersede integrated tissue-based diagnosis.

Moreover, several studies have shown that the use of AI to analyze histopathologic images and integrate multimodal imaging can facilitate the development of a new generation of multimodal prognostic models in neuro-oncology. Wang et al^22^ improved predictive performance for cancer diagnosis and prognosis using a pathology-based model with two complementary pretraining methods. Xu et al^26^ constructed a digital pathology foundation model and validated it across 26 distinct tasks, demonstrating its potential to assist clinical diagnosis and decision making. Xiang et al^27^ pretrained a vision language foundation model using large numbers of pathology images and pathology-related text annotations, integrating information from pathology images and clinical reports to enhance the accuracy and precision of cancer diagnosis and treatment. Ji et al^28^ combined histologic whole-slide images and genomic data to predict prognosis in eight cancer types, and Vanguri et al^29^ integrated medical imaging, histopathology, and genomics to construct a predictive model for immune checkpoint inhibitor response in patients with non–small cell lung cancer. In this study, we pretrained and validated our model using MRI as the input, demonstrating the clinical application potential of our model. However, considering the growing prospects of multimodal integration models, our model still has the potential to further improve its performance.

The SSL-pretrained UMBIF model achieved promising results across multiple neuro-oncology tasks, yet important limitations persist. First, it was trained primarily on conventional structural MRI sequences, without leveraging richer multimodal brain MRI acquisitions or integrating pathologic images, and the majority of patients were drawn from the European Union and the United States, reducing geographic representativeness. Second, key epidemiologic and clinical factors—such as age and sex—were not included, although they could provide informative covariates for neurosurgical and neurologic analyses. Third, the current scope is restricted to *IDH*, 1p/19q, and *MGMT*, and does not cover additional WHO-defining markers (eg, *ATRX*, *TERT* promoter, *EGFR* amplification, +7/–10 signature); it also depends largely on standard structural MRI rather than advanced sequences and lacks systematic incorporation of clinical covariates. Collectively, these issues motivate future extensions of UMBIF through broader, more diverse imaging cohorts, inclusion of additional modalities, and more flexible multimodal interaction strategies.

In conclusion, UMBIF is an MRI-based SSL framework with two-stage training on natural and 3D images that improves molecular pathology prediction and neuroimaging by reducing label dependence, enhancing generalizability, exploiting 3D structure, and outperforming supervised methods in data-limited settings while strengthening radiology-molecular integration and neurogenomics.
